# Extraction and Characterization of Collagen from Elasmobranch Byproducts for Potential Biomaterial Use

**DOI:** 10.3390/md18120617

**Published:** 2020-12-04

**Authors:** Manuel J. Seixas, Eva Martins, Rui L. Reis, Tiago H. Silva

**Affiliations:** 1 3B’s Research Group, I3Bs—Research Institute on Biomaterials, Biodegradables and Biomimetics, University of Minho, Headquarters of the European Institute of Excellence on Tissue Engineering and Regenerative Medicine, AvePark, Parque de Ciência e Tecnologia, Zona Industrial da Gandra, 4805-017 Barco, Guimarães, Portugal; manuelseixas94@gmail.com (M.J.S.); or eva.biotec@gmail.com (E.M.); rgreis@i3bs.uminho.pt (R.L.R.); 2 ICVS/3B’s–PT Government Associate Laboratory, Braga, Guimarães, Portugal

**Keywords:** elasmobranch byproducts, marine collagen, hydrogel, cartilage, tissue engineering, marine biomaterials

## Abstract

With the worldwide increase of fisheries, fish wastes have had a similar increase, alternatively they can be seen as a source of novel substances for the improvement of society’s wellbeing. Elasmobranchs are a subclass fished in high amounts, with some species being mainly bycatch. They possess an endoskeleton composed mainly by cartilage, from which chondroitin sulfate is currently obtained. Their use as a viable source for extraction of type II collagen has been hypothesized with the envisaging of a biomedical application, namely in biomaterials production. In the present work, raw cartilage from shark (*Prionace glauca*) and ray (*Zeachara chilensis* and *Bathyraja brachyurops*) was obtained from a fish processing company and submitted to acidic and enzymatic extractions, to produce acid-soluble collagen (ASC) and pepsin-soluble collagen (PSC). From all the extractions, *P. glauca* PSC had the highest yield (3.5%), followed by ray ASC (0.92%), ray PSC (0.50%), and *P. glauca* ASC (0.15%). All the extracts showed similar properties, with the SDS-PAGE profiles being compatible with the presence of both type I and type II collagens. Moreover, the collagen extracts exhibited the competence to maintain their conformation at human basal temperature, presenting a denaturation temperature higher than 37 °C. Hydrogels were produced using *P. glauca* PSC combined with shark chondroitin sulfate, with the objective of mimicking the human cartilage extracellular matrix. These hydrogels were cohesive and structurally-stable at 37 °C, with rheological measurements exhibiting a conformation of an elastic solid when submitted to shear strain with a frequency up to 4 Hz. This work revealed a sustainable strategy for the valorization of fisheries’ by-products, within the concept of a circular economy, consisting of the use of *P. glauca*, *Z. chilensis,* and *B. brachyurops* cartilage for the extraction of collagen, which would be further employed in the development of hydrogels as a proof of concept of its biotechnological potential, ultimately envisaging its use in marine biomaterials to regenerate damaged cartilaginous tissues.

## 1. Introduction

Fisheries and aquaculture have had an enormous increase since 1961 and in 2016 reached the maximum production of 171 million tons; at the same there is the urge to fight malnutrition and hunger worldwide [1]. Unfortunately, these fisheries are frequently unsustainable and some populations can suffer major damage from this overfishing [2] due to the generation of high quantities of by-products in fish-processing industries. Fish wastes can be either from discarded fish with low economic value, or wastes resulting from unused fish parts from fish processing industries such as skin, cartilage and viscera [3]. These fishery by-products are usually used for the production of fish meal [4], even though by-products can have an intrinsic value on the biomedical, nutraceutical, and biotechnological fields as sources of relevant compounds [5,6,7,8]. In researching marine fish, the subclass of elasmobranchs (composed mainly by sharks and rays) possesses a endoskeleton composed mainly by cartilage [9], which apparently can be a good source for collagen type II extraction [10,11]. Additionally, in 2011, cartilaginous fish recorded a global trade of around 120,000 tons, accounting only for meat values [12]. Coincidentally, sharks are habitually fished secondarily to tuna fisheries and their fillet processing results in cartilage as waste [13], similarly to what happens with rays although the latter has a greater economic value and food usage.

Cartilaginous tissues are an important tissue in most vertebrates, with a highly-hydrated extracellular matrix (ECM) [14], composed of collagens such as types I, II, IV, V, VI, IX, and XI [15], whereas type II collagen composes around 50–70% of the total dry weight [16,17]. Furthermore, it is composed of glycosaminoglycans, for example chondroitin sulfate, that can be combined with proteins forming proteoglycans, representing the second-most-present group of macromolecules in cartilage [18,19]. Cartilage is an avascular tissue and its main cells, the chondrocytes, are immobile when they reach a mature stage, hindering their proliferation and resulting in an inefficient healing in any damaged cartilaginous tissue. Nowadays, there is a panoply of ways to reduce symptoms of diseases like osteoarthritis, but the current methods are not capable of full recovery [20], demanding the establishment of new therapeutic methodologies. Presently, the development of biomaterials capable of mimicking cartilaginous ECM, and thus providing a matrix for chondrocytes to repair the damaged regions of cartilage, is being explored by many research teams, using a wide range of materials [21]. Currently, hydrogels can be produced using marine-origin raw-materials in wide applications such as alginates extracted from brown seaweed for brain tissue regeneration [22], seaweed-derived carrageenans isolated from red algae for drug delivery [23], or type I collagen extracted from shark skins as material for cartilage tissue engineering [24].

Type II collagen, being the greatest of the proteins present on the cartilage, is a gold standard in order to produce a biomaterial that can mimic cartilaginous tissues. However, this type of collagen is often extracted from land mammals [25] which can endanger consumers because of the risk of transmitting diseases such as bovine spongiform encephalopathy, or resulting in high cost to assure BSE-free materials, and raise ethical or religious concerns [16,26]. There is, thus, a pressure to find alternatives to mammal collagen, such as marine organisms, which have been receiving some consideration on the recent times [27]. Biomaterials combining type II jellyfish collagen, human stem cells, and TGF-β3 was demonstrated to enhance cartilage differentiation and repair [28]. Moreover, other components that are present on the ECM of the cartilage can be also considered, such as chondroitin sulphate (CS) that can be extracted by chemical and enzymatic hydrolysis from shark cartilage [29]. In addition, chondroitin sulfate (CS)-6 isomer (GlcA-GalNAc 6S) has a greater prevalence in blue shark (*Prionace glauca*) [30], and it is reported that intermediate values of CS-4 and -6 isomers affect cell proliferation and chondrogenic differentiation processes in marine species [31]. Furthermore, studies reported the use of hydrogel-produced collagen–chondroitin sulfate–hyaluronic acid that in part mimicked the composition of cartilage extracellular matrix, showing excellent cellular compatibility. Therefore, using chondrocytes can be a promising approach for the development of biomaterial for cartilage regeneration [32,33]. As noted, both collagen and chondroitin sulphate show remarkable capabilities for application in tissue engineering. Likewise, some of the previous studies show that this combination of biopolymers can be useful for cartilage regeneration when used as scaffold [34] or hydrogel biomaterial [32,35,36].

Therefore, the present study aims to valorize raw elasmobranch cartilage by-products, namely cartilage of shark and ray species, *Prionace glauca* (PG), *Zearaja chilensis,* and *Bathyraja brachyurops* from a Portuguese fish processing company, *P. glauca* is a shark that has worldwide distribution and is heavily fished [37,38], even in the Portuguese coastal waters, where the annual amount of landings has reached 440 tons [39], which leads to a “Near Threatened” status by the International Union for Conservation of Nature (IUCN) [40]. Therefore, it important to find sustainable ways to explore such species, including with the valorization of its by-products. This species was reported to be unhealthy for human consumption in some regions [41], but its potential as source of valuable compounds, namely type I collagen for biomaterial production [42], chondroitin sulfate [29], which is already used on hydrogels for cartilage tissue engineering [43], besides antioxidants [44], and antitumoral substances [45] is well recognized. In turn, rays species were identified as a mixture of *Z. chilensis* and *B. brachyurops,* presenting a “Vulnerable” [46] and “Least Concern” [47] status by the IUCN, respectively. These species are mostly located in the South Atlantic and to date there are no reports of studies regarding an eventual biomedical benefit. In the Portuguese coastal waters, there are no reports of catches of these species, but it is estimated that the annual amount of rays landings has reached 1100 ton [39]; so, exploitation for human consumption as food results in significant quantities of such by-products as cartilage and skin. In parallel to the valorization of by-products, and relying on the results from these efforts, it is important to bring awareness to the global policy-makers that these organisms present a richer value to humankind, which demands intense supervision of their sustainable exploitation in order to preserve the marine ecosystem.

The objective of the present work is the study of a valorization strategy for the above mentioned elasmobranchby-products based on the isolation and characterization of collagen and an evaluation of the formation of hydrogels using the produced biopolymers envisaging a future application in cartilage tissue engineering. While most biomaterials produced with elasmobranch byproducts focus on skin and bone tissue engineering, by using collagen extracted from these organisms’ skin, this work aims to evaluate the possibility of using collagen extracted from their cartilage. This byproduct is mainly explored for the isolation of chondroitin sulfate and there are only few published works on the extraction of collagen and its usability for biomaterial production. Collagen was extracted using acidic and enzymatic methodologies and the obtained biopolymers were characterized by electrophoresis, amino acids quantification, FTIR, thermal analysis, and circular dichroism. The collagen produced with higher yield was further combined with chondroitin sulfate for the production of hydrogels, characterized by rheology.

## 2. Results and Discussion

### 2.1. Collagen Extraction Yield

Beginning from the standpoint of envisaging the use of the extracted collagen in the biomedical industry, it is important to produce high quality materials, with preserved properties complying with application requirements together with high purity. Using both methodologies, it was possible to obtain marine collagens from shark and ray cartilages and, according to the results displayed in Table 1, PG PSC was clearly the one produced most efficiently (3.5%). Typically, shark collagen extractions have a greater yield in enzymatic extractions than the acidic ones [48], whereas PG ASC follows this trend (0.15%). In a previous study with different species, other researchers reported lower extraction yields from shark than from ray species [11]. Even though there are not a large number of results for type II collagen, in some studies it has a higher yield than all of the other extractions we performed. This can be due to differences in the extraction methodology, namely regarding the efficiency of collagen extraction from cartilage biomass or collagen precipitation and retrieval from extraction solution. Besides, the yield of extraction can be also affected by the water content of the biomass (the values used are regarding wet cartilages), which can be very variable between studies [49]. Additionally, the extractions performed in the present work have a lower yield than the ones achieved by others with shark cartilage extractions, which have reached values as high as 1% for acidic extraction and 10% for the enzymatic extractions [48]. These differences can be related to the mixture of species present on the ray batch or the specificity of each species used in the different studies. The utilized methodology can still be optimized, obtaining a higher yield that would be important in a scaled-up process, to be used on an industrial level.

Besides the yield of extraction determined from the weight of extract obtained, it is also important to understand the purity of the extract, which may be assessed by determining collagen contents based on hydroxyproline (HPro) quantification. Typically, this is made assuming as reference a HPro content of 12.5% (*w*/*w*) in collagen [50,51]. However, this reference value relates to mammalian collagen and it is known that fish collagens have a minor amount of this amino acid [52,53,54]. In fact, for cartilage of *Prionace glauca*, HPro content was determined as 7.5% [55], which was used as a reference value in the present work. The estimated values of collagen content in the produced extracts are given in Table 1. It is possible to see the high purity of PG ASC (93%) and PG PSC (91%), while the value obtained for Ray PSC suggests a significant presence of non-collagenous proteins in the extract.

### 2.2. SDS-PAGE

**Figure 1:**
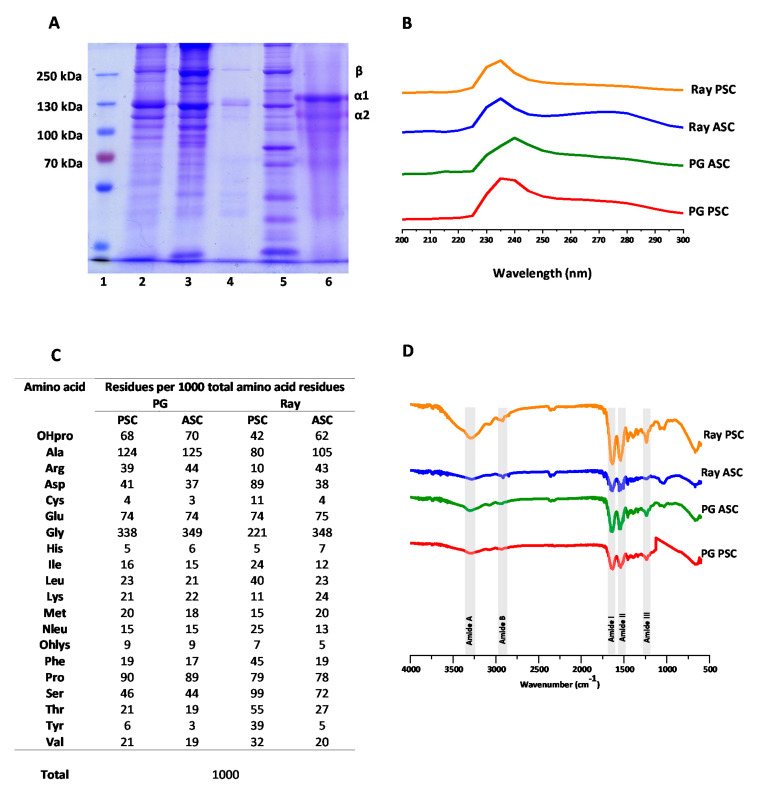
SDS-PAGE, UV–VIS, amino acid determination, and FTIR analyses of elasmobranch collagens. (**A**) Electrophoresis analysis of collagen extractions: 1, protein marker; 2, *Prionace glauca* (PG) pepsin-soluble collagen (PSC); 3, PG acid-soluble collagen (ASC); 4, ray PSC; 5, ray ASC; and 6, type II collagen from chicken sternum. (**B**) Absorption spectrum of PG PSC, PG ASC, ray PSC, and ray ASC. (**C**) Amino acid compositions of PSC and ASC collagens from shark cartilage and ray cartilage (residues per 1000 total amino acid residues). (**D**) FTIR spectra of the performed PG PSC, PG ASC, ray PSC, and ray ASC.

The collagenous extracts were collectively screened on an electrophoretic gel, together with a reference type II collagen from chicken sternal cartilage (Sigma Aldrich, Gillingham, Dorset, UK), with illustrative results being depicted in Figure 1A Dissecting the obtained SDS-PAGE profiles, it was possible to observe a bright band around the 250 kDa which is associated with two alpha bands linked to each other—generating a so-called beta band—indicating the presence of crosslinking bonds between alpha chains. Moreover, other bands were observed between 130 and 100 kDa, corresponding to the different alpha chains—type I collagen is composed of two α1(I) chains and one α2(I) chain, whereas type II collagen is composed of three α1(II) collagens [56]. Since the brighter bands on all the collagen extractions were from the α1 chains, it is possible to extrapolate that the collagenous extractions contained collagen type I and type II, which is similar to what was found in other studies with shark species [57]. These bands on the electrophoretic pattern of the elasmobranch collagens were revealed to be similar to the bands evidenced by the reference type II collagen and consistent with the results obtained in other collagen extraction studies [10,58,59]. The difference versus the reference collagen was on the inferior position of the bands, corresponding to lower molecular weight that can be due to different amino acid sequences (non-collagenous proteins) or collagen peptides resulting from partial hydrolysis. Considering the high purity of the extracts depicted in Table 1, the presence of collagen hydrolysates is the most probable, excepting the case of ray PSC which exhibited a low purity and could have contained non-collagenous proteins or other compounds not detected by SDS-PAGE. Moreover, enzymatic extractions overall have a higher molecular weight than the acidic ones.

### 2.3. UV–VIS Spectroscopy

UV–visible photospectroscopy was used to estimate the protein purity of the extracts, based on the theory that the characteristic amino acids of collagen result in a maximum absorbance around 230 nm [60], while other proteins absorb UV radiation around 280 nm [61]. The illustrative spectra of collagenous extracts are shown in Figure 1B and demonstrate that PG PSC, ray PSC, and ray ASC peaked at 235 nm and PG ASC at 240 nm, compatible with the presence of collagen, in accordance with the results from the previous analyses. Moreover, overall, there was a low amount of other proteins in the extracts as there were no significant signals at 280 nm, with exception of ray PSC, confirming that the used methodology is capable of removing other non-collagenous proteins and rendering barely-pure extracts, in accordance with other studies [58,59]. In the case of ray PSC where a broad band is visible around 280 nm, other proteins should be also present, suggesting that the correspondent procedure was not successful on the production of a pure collagen extract, in accordance with the results described before for the SDS-PAGE, where the bands profile is significantly different from the other assessed samples.

### 2.4. Amino Acid Analysis

The amino acid contents of the elasmobranch cartilage extracts are depicted in Figure 1C, as the molar ratio of a given amino acid in respect to 1000 total amino acid residues (the approximate number of amino acids in each collagen alpha chain). The three more abundant amino acids were glycine, alanine, and proline for PG PSC, PG ASC, and ray ASC samples, with Gly accounting for about one third of the amino acid residues, which correlates very well with the known composition of collagen characterized by sequence repetitions of the triplets Gly-X-Y, with X being often proline and Y being often hydroxyproline [62]. Moreover, the obtained results are also similar with the ones obtained with other elasmobranch species [10]. In turn, the major amino acids present in ray PSC extract were glycine, serine, and aspartic acid, which may be explained by the inefficient removal of pepsin used in the enzymatic treatment that had a high composition of aspartic acid and serine [63], confirming the impurity of this extract as suggested earlier by UV spectroscopic analysis.

Furthermore, excluding ray PSC, it is worth noticing that the relative abundance of proline and hydroxyproline, with hydroxyproline resulting from the hydroxylation of proline, is a characteristic post-translation modification of collagen proteins that enhances the stabilization of the triple helix conformation [64] and is used as marker of the presence of collagen in proteic extracts and can be even used for collagen quantification [65,66]. In the case of ray PSC, hydroxyproline was also present, confirming the presence of collagen in the extract, but its lower ratio, together with the contents of other amino acids, supports the presence of other proteins in the extract. The degree of hydroxylation of elasmobranch collagens was 43%, 44%, 35%, and 44% for PG PSC, PG ASC, ray PSC, and ray ASC, respectively, similar to degree of hydroxylation (47.29%) reported in collagen from the cartilage of Amur sturgeon [67].

### 2.5. Fourier Transform Infrared (FTIR) Spectroscopy

FTIR analysis can explore some of the chemical characteristics of the extracts, as collagen is usually portrayed by the presence of five amide peaks—amide A, amide B, amide I, amide II, and amide III [10,27,61]. The obtained spectra (Figure 1D) showed these representative signals. Firstly, a soft peak related with the amide A band, was associated with the frequencies of N–H stretching that usually ranges from 3310 and 3270 cm^−1^ [68,69], was here observed at lower wavenumber, which is a good indicator of a stronger hydrogen bond [70]. Next, the amide B peak was observed in the range between 3080 and 2889 cm^−1^ related with N–H stretching [71]. There was a slight variation on the wavenumber obtained between shark and ray extracts, contrary to that observed when comparing acid and enzymatic extracts where the values did not differ significantly. Related to the secondary amides, amide I signal results from C=O stretching on proteins [72], characteristically observed as a strong peak around 1600 cm^−1^. Likewise, the amide II peak generally ranges between 1580 to 1500cm^−1^ representing a N–H bending [73], which was observed for all extracts. Finally, an amide III signal that indicated N–H bending and C–N stretching [74] was observed in the characteristic range from 1200 to 1350 cm^−1^ [75].

### 2.6. Differential Scanning Calorimetry (DSC)

**Figure 2:**
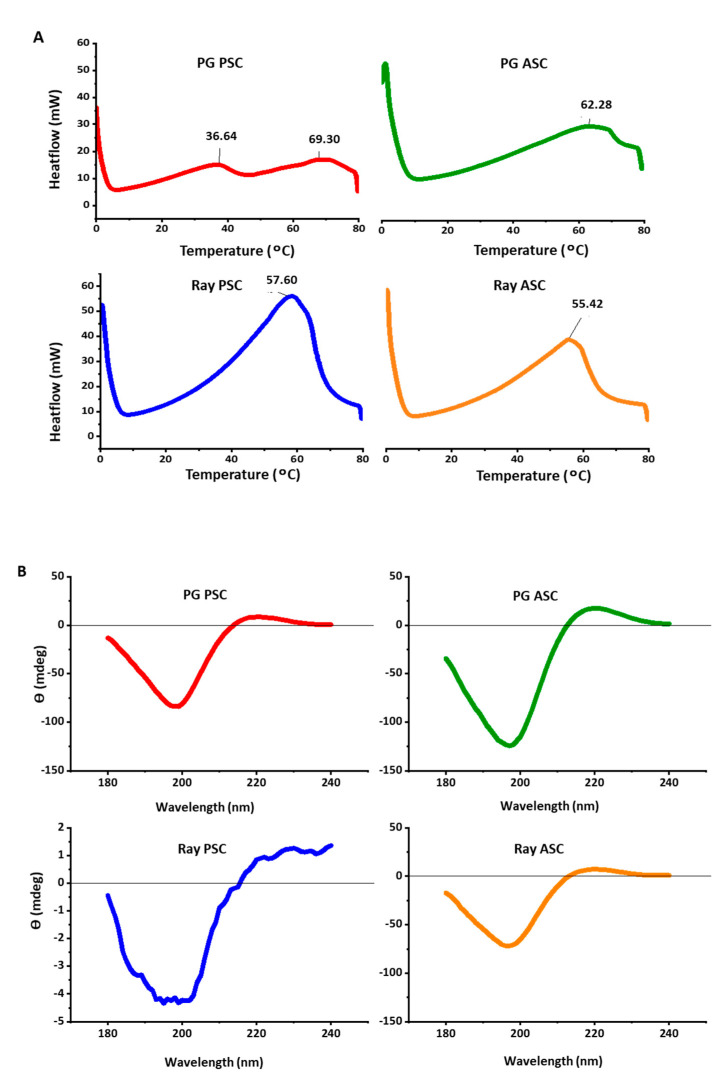
Differential scanning calorimetry (DSC) and circular dichroism (CD) results of elasmobranch collagens. (**A**) DSC and (**B**) CD spectra of PG PSC, PG ASC, ray PSC and ray ASC collagens obtained at 4 °C.

DSC was used to estimate the temperature at which collagen denatured, as proteins tend to unfold while absorbing heat [76]. Considering the ultimate goal of application of collagen, it is important to understand its thermal stability and, particularly considering the biomedical arena, the performance at 37 °C (human basal temperature) is particularly crucial [77]. The obtained thermogram results (Figure 2A) are characterized by bands at temperatures as high as 60 °C, not compatible to the low values (<30 °C) commonly described for marine collagens, even considering possible variations with the age and natural environment of the individual organism [78]. This may be an indication that the phenomenon being detected does not correspond to collagen denaturation but probably to collagen melting or thermal gelation, as it is being performed with dry (freeze-dried) samples. Besides, the variability of peak temperature may be accounted for not to species variation, but to experimental artifact, ultimately suggesting that the used methodology is not the most appropriate to assess collagen denaturation. In any case, all the extracts in the dry state withstood the temperature of the human body and may be considered for further biomedical prospect.

### 2.7. Circular Dichroism (CD)

In order to evaluate the secondary structure of the extracted collagens, and in particular to confirm that the extraction process was not detrimental and did not denatured the proteins, the produced extracts were characterized by CD. Denaturation would correspond to the loss of the characteristic triple helix conformation, with dramatic effects on the performance of the extracted biomolecules [79,80]. The spectra obtained with PG PSC, PG ASC, and ray ASC, illustrated by the graphs in Figure 2B, showed a negative peak around 200 nm, a crossover point on the *x*-axis around 210 nm, and a positive peak around 220 nm, which is representative of the presence of preserved triple helix [81,82]. In the case of ray PSC, an irregular spectra was obtained, with a lower signal to noise ratio, although it is possible to see the pattern related to the triple helix, both the negative and positive peaks; this is in accordance with the differences observed with the other analyses abovementioned, all suggesting that this extract was not produced as successfully, with a significant amount of impurities being present.

### 2.8. Hydrogel Stability

With the goal of producing a suitable hydrogel, and in particular envisaging an application as a 3D-cell-culture template in biomedical approaches, it is important that it achieves its macro conformation characterized by a cohesive polymer matrix, structurally stable at 37 °C. Within the performed extractions, besides ray PSC, all collagens had similar properties (purity, conformation preserved, and thermal stability). Since PG PSC presented the higher yield it was the selected extract to produce the hydrogels. PG PSC was combined with chondroitin sulfate using different formulations, utilizing EDC (1-ethyl-3-(3-dimethylaminopropyl)carbodiimide hydrochloride) as the crosslinker, varying the temperature and different incubation times, as detailed in Table 2. The success of biomaterial production was addressed by macro visualization (apparent cohesiveness), manipulation with spatula and forceps and structural stability upon incubation in cell culture medium at 37 °C. The obtained results showed that a higher concentration of EDC performed more effective reticulations, but it can be toxic if the excessive EDC could not be completely washed out and removed [43]. Furthermore, increasing the temperature and reaction time proved to be beneficial for the reticulations, even though incubation at −20 °C can increase the gel porosity due to ice crystal formation that did not happened at 4 °C (this can be promoted afterwards, if needed, by freeze-drying the samples post reticulation) [83]. Lastly, the solvent change from acetic acid to hydrochloride acid offered a breakthrough, enabling the production of hydrogels capable of withstanding 37 °C while keeping their physical integrity. This may be explained by the stronger acid character of HCl that would result on a better dissolution and might increase the availability of the substances for crosslinking.

**Figure 3:**
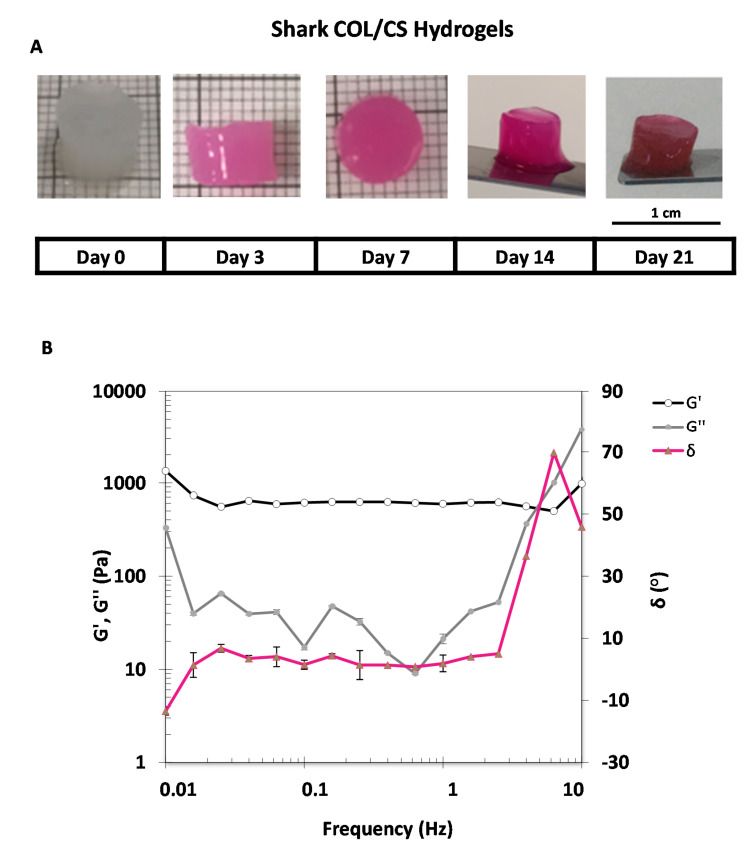
(**A**) Stability of the hydrogels using the formulation “M” upon incubation in Dulbecco’s Modified Eagle’s Medium (DMEM) from 0 to 21 days at 37 °C; (**B**) variation of elasticity modulus G’ and viscosity modulus G’’. Phase angle (°) in an increasing frequency sweep assay.

Overall, the hydrogels produced utilizing the formulation “M” were able to maintain their conformation for 21 days and exhibited the better performance as far as cohesiveness is concerned, as illustrated by the images in Figure 3A, demonstrating the manipulability of the biomaterial. It is worth noting that, although being possible to produce cohesive hydrogels with only PG PSC collagen (formulation N as reference biomaterial), its stability in aqueous media was significantly lower when compared with other formulations (namely L and M), thus showing the contribution of chondroitin sulfate to reinforcing the stability of the resulting hydrogels.

### 2.9. Rheology of Collagen/Chondroitin Sulfate Hydrogel

After achieving a formulation capable of producing hydrogels that maintain physically integrity at the human basal temperature, it was pertinent to understand the mechanical properties of the biomaterial, particularly the response to shear stress. In this regard, the selected hydrogel was submitted to rheological analysis, where the oscillatory sweeps from 0.01 to 5 Hz enabled the assessment of the variation of G’ (elasticity modulus) and G’’ (viscosity modulus) during the oscillation tests, whereas the material will had an elastic solid conformation if G’ was higher than the G’’, with stiffer hydrogels being characterized by higher G’ [84]. In accordance, the hydrogel “M” showed a solid elastic conformation presenting a G’ ranging near 600 Pa, higher than the G’’ till 4 Hz and a phase angle of 36.5°, that seemed to be the rupture condition of the hydrogel, since it lost its solid conformation (G’’ > G’). Furthermore, the hydrogels showed a stable elastic modulus during the sweep till the rupture point. Nevertheless, the G’ recorded on other studies was still short compared to other hydrogels being proposed for cartilage substitution [85,86], and much different to the human knee cartilage tissue that can withstand shear frequencies ranging from 1 to 1000 Hz on a daily basis [87]. Based on the exhibited properties, this shark collagen/chondroitin sulfate hydrogel may be idealized as a regenerative biomaterial instead of replacing the damaged cartilage tissue (substitutive approach). This envisaged application requires the absence of any toxicity (namely caused by cross-linking) and the capability to support the culture of chondrocytes for their proliferation and synthesis of new extracellular matrix components towards new cartilage formation [88,89]. This will be the object of further studies in our laboratory.

## 3. Materials and Methods

### 3.1. Raw Materials

Raw elasmobranch by-products composed mainly by shark (*Prionace glauca*, PG) and ray (*Zearaja chilensis* and *Bathyraja brachyurops*) cartilages, were kindly offered by NIGEL Lda. (Peniche, Portugal) and kept frozen until further use.

### 3.2. Collagen Extraction

**Figure 4:**
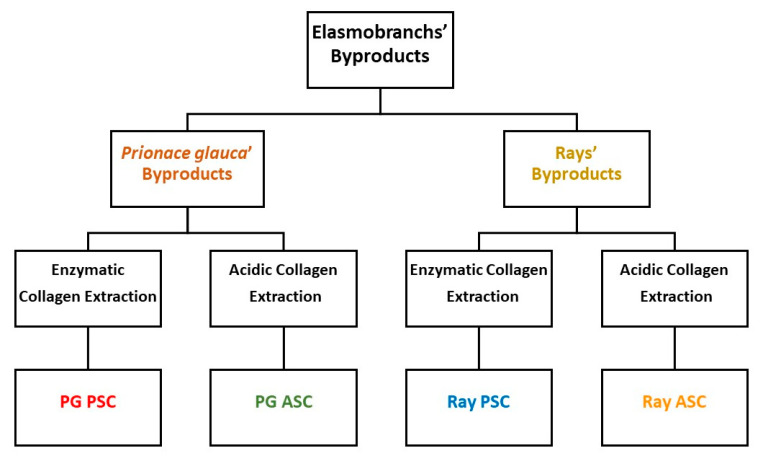
Schematic representation of the extraction processes to obtain collagen from shark and ray cartilages.

The collagens were extracted by the methodology described in the literature [48] with slight modifications, obtaining four marine collagens isolated from shark and ray cartilage byproducts (Figure 4). All the extraction procedures were carried out at 4 °C. The shark and ray cartilages were cleaned of muscle debris and washed with distilled water to remove other impurities. Afterwards, cartilages were submerged in 0.1 M NaOH (Sigma-Aldrich) (1:10 *w*/*v*) for 6 h, with stirring, changing solution every 2 h. After washing with distilled water until neutrality, the biomass was decalcified with 0.5 M EDTA (Sigma-Aldrich) (1:4 *w*/*v*) for one week, and again washed with water until neutrality, cut in smaller pieces and divided in batches to proceed separately with acid and enzymatic extractions. For acid extraction, biomass was treated with 0.5 M acetic acid (1:15 *w*/*v*) for 48 h, with stirring. After removing the remaining cartilage by filtration, the resultant solution was centrifuged for 30 min at 20,000× *g*, whereas the supernatant was collected, containing acid-soluble collagen (PG or ray ASC) and the pellet was discarded. For enzymatic extraction, biomass was treated with 0.5 M acetic acid (1:15 *w*/*v*) and 1% pepsin (Sigma-Aldrich) (1:6 *w*/*v*) for 48 h, with stirring. The resultant solution was centrifuged for 30 min at 20,000× *g*, collecting the supernatant containing pepsin-soluble collagen (PG or ray PSC). Collagen from each of the resultant supernatants was precipitated with a 2.6 M NaCl solution in 0.05 M Tris-HCl buffer solution with pH 7.5 (1:2 *v*/*v*), overnight, and retrieved by centrifugation at 20,000× *g* during 1 h. The obtained pellets were resuspended in 0.5 M acetic acid and dialyzed against 0.1 M acetic acid for 2 days, 0.02 M acetic acid for 2 days, and water until pH 7 (renewing the solution every day). Finally, collagen was frozen at −80 °C, lyophilized, and stored at room temperature until further characterization.

The extraction yield was calculated for all the four extractions (PG ASC, PG PSC, ray ASC, and ray PSC) as the ratio between the weight of lyophilized collagen and the wet weight of cartilage from each of the elasmobranch species, according to the following equation.

(1) $$\mathrm{Yield}\;\mathrm{of}\;\mathrm{collagen}\;\left(\%\right)=\frac{\mathrm{Weight}\;\mathrm{of}\;\mathrm{collagen}\;\left(g\right)}{\mathrm{Weight}\;\mathrm{of}\;\mathrm{dry}\;\mathrm{cartilage}\;\left(g\right)}\times 100$$

### 3.3. Collagen Characterization

To evaluate the purity of the extracts and the molecular weight of the protein fractions, a sodium dodecyl sulfate-polyacrylamide gel electrophoresis (SDS-PAGE) was performed using the SDS-PAGE preparation kit from Sigma-Aldrich and run on a Biorad Mini Protean II System. The lyophilized collagen was dissolved in 0.5 M acetic acid (1 mg·mL^−1^), mixed with loading buffer (1:1 *v*/*v*) and heated for 10 min at 95 °C in order to denature the protein. The SDS gel was composed of 7.5% separation gel and 3% stacking gel, where the latter was loaded with 4 μL protein ladder (PageRuler Plus Prestained Protein Ladder, 10 to 250 kDa—ALFAGENE), 20 μL from each sample, PG PSC, PG ASC, ray PSC, and ray ASC, compared to a type II standard sternal collagen from chicken (Sigma-Aldrich). The samples were run at 90 V and the gel was then stained using the Coomassie solution (0.500 g Coomassie Brilliant Blue G-250 (Biorad, Hercules, CA, USA), 500 mL methanol, 100 mL acetic acid, and 400 mL deionized water) for 30 min with stirring and the stain excess was removed using a destain solution (5% methanol and 7% acetic acid).

Additionally, collagens were also characterized by UV-Vis spectroscopy, dissolving them in 0.5 M acetic acid (1% *w*/*v*) and analyzed on a microplate reader (Synergy™ HT—Biotek, Winooski, VT, USA) ranging from 200 to 300 nm.

Amino acid contents were assessed using Biochrome 30 apparatus (Biochrome Ltd., Cambridge, UK) whereas the extracts were fully hydrolyzed and split by an ion-exchange column, later derived by ninhydrin and analyzed at the wavelengths of 440 and 570 nm. An internal standard of norleucine was used to determine the concentration of amino acids in the sample. Hydroxylation of elasmobranch collagens was calculated, using Equation (2), whereas pyrrolidine amino acid content was the sum of hydroxyproline (OHPro) and proline (Pro) amino acids (chemically, amino acids derivatives of pyrrolidine).

(2) $$\mathrm{Hydroxylation}\;\left(\%\right)\;=\frac{\;\mathrm{OHPro}\;\mathrm{content}}{\;\mathrm{pyrrolidine}\;\mathrm{amino}\;\mathrm{acid}\;\mathrm{content}}\;\times \;100$$

Moreover, Fourier-transform infrared (FTIR) spectroscopy in attenuated total reflectance (ATR) mode, using freeze-dried collagen samples, was performed (IRPrestige 21—Shimadzu, Kyoto, Japan) between 4000 and 500 cm^−1^ with a resolution of 2 cm^−1^, with each spectrum being the average of 32 scans, to evaluate the presence of collagen’s characteristic chemical bounds/groups.

Furthermore, to determine the denaturation temperature of the extracted collagens, the samples were dissolved in 0.5 M acetic acid at a concentration of 3% and analyzed by a differential scanning calorimeter (DSC Q100—T. A. INSTRUMENTS, New Castle, DE, USA), measuring the heat flow on when heating from 0 °C to 80 °C, at a scanning rate of 10 °C·min^−1^.

To evaluate the protein conformation of the extracted collagens, circular dichroism (CD) analysis was performed (J1500 CD spectrometer, Jasco, Tokyo, Japan), using a quartz cylindrical cuvette with a path length of 2 mm. Samples were dissolved at 0.1 mg·mL^−1^ in 50 mM acetic acid, at 4 °C, and 600 µL aliquots were added to the cuvette, with CD spectra (average of three scans) being obtained from 180 to 240 nm, at a scan rate of 50 nm·min^−1^.

### 3.4. Hydrogel Production

PG PSC was the collagen selected to produce the hydrogels. This material was dissolved in 0.5 M acetic acid or 0.01 M HCl, at 3% (*w*/*v*) or 6% (*w*/*v*) and combined with shark chondroitin sulfate (Sigma) at different ratios (80:20, 70:30, and 60:40) in order to mimic the human articular extracellular matrix macromolecular composition [90]. The resulting mixtures were placed into a silicone mold and EDC was added and let under reaction at 4 °C or −20 °C, during 4 h or 72 h, to promote chemical crosslinking.

### 3.5. Hydrogel Characterization

#### 3.5.1. Stability Test

The structural stability of the reticulated hydrogels was evaluated upon incubation (BE500—Memmert, Schwabach, Germany) in cell culture media Dulbecco’s Modified Eagle’s Medium (DMEM), at 37 °C, during up to 21 days and observing its physical integrity.

#### 3.5.2. Rheology

Rheological analysis of the selected hydrogel formulation was performed using a rheometer (Kinexus Prot, Malvern, Worcestershire, UK), acquiring the data with rSPACE software, utilizing a plate of 20 mm diameter and a geometry of 8 mm. Oscillatory experiments at frequency ranging between 0.01 and 5 Hz, with a shear strain of 0.04. The experiments were performed at 37 °C, utilizing a dodecane cover to maintain the temperature and prevent water loss.

## 4. Conclusions

The raw cartilage of elasmobranchs showed potential for collagen extraction, which can be an added value product from the valorization of byproducts from fish processing industries, with clear environmental and economic benefits. It was possible to produce collagen extracts with preserved triple helix conformation, with the methodology combining acid extraction with pepsin digestion to solubilize PSC from cartilage of blue shark (*P. glauca*) rendering the higher yield of 3.5% and a high purity extract (91% of collagen contents). Moreover, the hydrogel produced using PG PSC in combination with shark-derived chondroitin sulfate, as proof of concept of collagen processability, exhibited a cohesive polymeric matrix, with this marine biomaterial being here proposed to be tested in the future as a template for cartilage regeneration.

Besides the relevance for the biotechnology community, this work also adds to the need of elasmobranchs preservation and sustainable exploitation. Sharks, often listed as endangered species by the International Union for Conservation of Nature (IUCN), have an incredible potential of enhancing the human welfare, besides their preponderant role in the ecosystem and should be properly conserved, in accordance with the SDG 14 established by the UN 2030 Sustainability Agenda.

## Figures and Tables

**Table 1:** Extraction yield and hydroxyproline and collagen content from the collagen extractions of blue shark and ray cartilage in acid and enzymatic extractions.

Elasmobranch Collagens	Extraction Yield (%)	Ohpro in Extract (%)	Collagen Content (%)
PG PSC	3.5	6.8	91
PG ASC	0.15	7.0	93
RAY PSC	0.50	4.2	56
RAY ASC	0.92	6.2	83

**Table 2:** Hydrogel formulations varying the solvent, the collagen (COL), and chondroitin sulfate (CS) concentrations and incubation time and temperature. The respective outcomes, namely regarding reticulation and stability, are also given, where “+” represents 1 day; “++” represents a week, and every “+” next to that is one more week at 37 °C; “−” refers to the formulation not being stable for 1 day.

Designation	Solvent	Ratio COL/CS (m/m)	EDC (mg/mL)	Incubation Time (h)	Incubation Temperature (°C)	Reticulation	Stability (37 °C)
A	0.5 M AcOH	70/30	0.57	4	−20	Low cohesion	
B	0.5 M AcOH	60/40	0.57	4	−20	Low cohesion	
C	0.5 M AcOH	80/20	0.57	4	−20	No	
D	0.5 M AcOH	70/30	0.57	4	−20	No	
E	0.5 M AcOH	60/40	0.57	4	−20	Low cohesion	
F	0.5 M AcOH	70/30	0.96	4	−20	Low cohesion	
G	0.5 M AcOH	60/40	0.96	4	−20	Low cohesion	
H	0.5 M AcOH	70/30	4.79	72	4	High cohesion	−
I	0.5 M AcOH	60/40	4.79	72	4	High cohesion	−
J	0.5 M AcOH	70/30	9.58	72	4	High cohesion	+
K	0.5 M AcOH	60/40	9.58	72	4	High cohesion	+
L	0.01 M HCl	60/40	4.79	72	4	High cohesion	++
M	0.01 M HCl	60/40	9.58	72	4	High cohesion	++++
N	0.01 M HCl	100/0	9.58	72	4	High cohesion	+
